# A Systematic Mapping Review of 525 Randomised Controlled Trials on Pharmacological and Non‐pharmacological Interventions for People Living With Dementia and Their Formal and Informal Carers in Chinese Communities

**DOI:** 10.1002/gps.70240

**Published:** 2026-07-16

**Authors:** Cheng Shi, Maximilian Salcher‐Konrad, Jacky C. P. Choy, Dara Kiu Yi Leung, Jingxing Song, Adelina Comas‐Herrera, David Mcdaid, Martin Knapp, Gloria Wong

**Affiliations:** ^1^ School of Graduate Studies Lingnan University Hong Kong China; ^2^ Institute of Policy Studies Lingnan University Hong Kong China; ^3^ Pharmacoeconomics Department WHO Collaborating Centre for Pharmaceutical Pricing and Reimbursement Policies Austrian National Public Health Institute (Gesundheit Österreich GmbH/GÖG) Vienna Austria; ^4^ Department of Health Policy Care Policy and Evaluation Centre (CPEC) London School of Economics and Political Science London UK; ^5^ Department of Social Work and Social Administration The University of Hong Kong Hong Kong China; ^6^ Department of Social Work The Chinese University of Hong Kong Hong Kong China; ^7^ School of Psychology and Clinical Language Sciences University of Reading Reading UK

**Keywords:** carers, Chinese communities, dementia, dementia intervention, systematic review

## Abstract

**Objectives:**

Aligned with the World Health Organization's call for enhanced dementia research to inform national strategies, this systematic mapping review identifies critical gaps in the evidence base for Chinese populations. Representing over 20% of people living with dementia globally, this group remains underrepresented in the Western‐centric research that currently guides best practices. This is the first study that comprehensively reviews evidence on dementia‐related interventions trialled among participants identified as Chinese worldwide across both Chinese and English literature. It addresses critical gaps in previous Anglo‐centric reviews by capturing culturally specific interventions and assessing the evolving evidence landscape for Chinese population.

**Methods:**

Following PRISMA guidelines, we systematically searched two widely used Chinese databases (China National Knowledge Infrastructure, Wanfang Data) and 12 English bibliographical databases (MEDLINE, EMBASE, PsycINFO, CINAHL Plus, Global Health, WHO Global Index Medicus, Virtual Health Library, Cochrane CENTRAL, Social Care Online, BASE, MODEM Toolkit, Cochrane Database of Systematic Reviews) to identify randomised controlled trials (RCTs) published between 2008 and 2020 (registered on PROSPERO: CRD42019134135). Dual‐language teams employed culturally sensitive strategies to maximise coverage of regionally prevalent interventions. Risk of bias was assessed using version two of the Cochrane risk‐of‐bias tool for randomised trials. A narrative approach was used to review and assess the current landscape of dementia intervention research in Chinese communities.

**Results:**

We identified 183,277 records across Chinese and English databases and included 525 unique RCTs for synthesis (93% published in Chinese). The most commonly studied interventions were multicomponent interventions (35.2%), followed by pharmacological (29.5%), non‐pharmacological (18.3%), and traditional Chinese medicine (TCM; 14.5%) interventions. 62.7% of multicomponent RCTs were those combining TCM with Western drugs. Cognitive outcomes were measured in 86.9% of RCTs and functional outcomes in 60.2%, while only 3% evaluated quality of life (QoL) and 1% carer‐related outcomes. Most trials (91.8%) were hospital‐based. Over 80% of included RCTs were assessed as having overall ‘some concerns’.

**Conclusions:**

Despite rapid growth of dementia intervention research in Chinese communities, evidence remains skewed towards hospital‐based trials conducted predominantly in mainland China, with a focus on cognitive and functional outcome. High‐quality evidence is urgently needed to rebalance research priorities and address critical knowledge gaps in improving QoL, carer support, and community‐based interventions. Our finding that most trials were published in Chinese involving TCM illustrates the value of integrating non‐English literature in global evidence synthesis, especially for identifying culturally specific interventions common in lower‐ and middle‐income countries.

## Key Points

- This bilingual systematic mapping review identified 525 dementia‐related randomised controlled trials involving Chinese participants, the vast majority of which were published in Chinese and conducted in mainland China.
- Multicomponent interventions, often combining traditional Chinese medicine with Western pharmacological treatments, were the most frequently studied, while non‐pharmacological and carer‐focused interventions remained relatively underdeveloped.
- Most trials were hospital‐based and prioritised cognitive and functional outcomes, with very few studies addressing quality of life, economic outcomes, or carer well‐being.
- Overall methodological quality raised concerns, highlighting an urgent need for rigorously designed community‐based trials that broaden outcome domains and better support people living with dementia and their carers in Chinese communities.

## 1 Introduction

Globally, around 55 million people are currently living with dementia in 2020, a number projected to rise to 139 million by 2050 [1]. A large‐scale national study suggested that about 15.07 million persons aged 60 and above live with dementia in mainland China [2], accounting for 27.4% of global dementia cases. This makes China the country with the worlds’ largest population affected by dementia. Beyond China, there are also estimated 50 million overseas ethnic Chinese [3], comprising both emigrants and their descendants, who represent a significant demographic potentially influenced by the condition. The substantial size of these populations highlights the importance of conducting dedicated research on dementia intervention relevant to Chinese populations. Timely and effective interventions are crucial to delay disease progression and improve the quality of life (QoL) of people living with dementia and their carers. The World Health Organization (WHO) emphasises the importance of a national dementia strategy and includes publishing a national strategy as one of the proposed actions of the global action plans by 2025 [4]. In Western countries, some effective interventions have been identified and integrated into routine care. For example, Cognitive Stimulation Therapy (CST) is recommended by the National Institute for Health and Care Excellence (NICE) in England and included as an essential standard of the UK Memory Services National Accreditation Programme (MSNAP). The development of practice standards and national strategies for dementia in non‐Western societies, including Chinese communities, must be informed by locally relevant evidence where available [5]. Understanding the current state of dementia intervention research in Chinese communities is therefore critical to guiding policy design and implementation.

Over the past decade, a growing body of evidence has emerged on the effectiveness of interventions aimed at improving the well‐being of individuals affected by dementia, including both pharmacological and non‐pharmacological approaches. However, the majority of evidence has been generated in Western countries [6, 7]. The relevance of effective interventions identified in the West may be questionable for Chinese populations due to potential cultural differences and pharmacogenetic factors. In this review, ‘Chinese populations’ refers to individuals identified as being of Chinese ethnicity or heritage in the original studies, recognising that such populations are culturally and contextually heterogeneous across regions and health systems. Cultural adaptation frameworks [8] emphasise that dementia interventions must align with local health beliefs, family structures, and practices —areas where Western‐developed models often fall short in Chinese contexts. Research has shown that Chinese carers' experiences are deeply shaped by cultural values such as filial piety, collective family responsibility, and stigma surrounding dementia, which influence both their willingness to seek help and their preferred sources of support [9]. Service providers also highlight challenges unique to Chinese families, including limited community resources, family conflict in decision‐making, and the need for trust‐building and culturally sensitive education [9]. Existing systematic reviews have paid limited attention to evaluating intervention effectiveness specifically for Chinese populations with dementia or mild cognitive impairment (MCI). While several reviews have examined specific intervention types [10, 11, 12], only two have focussed on interventions for Chinese carers of people with dementia [13, 14]. One synthesised the characteristics of dementia caregiving interventions for Chinese families [13], while the other reported mixed efficacy across outcomes [14]. Collectively, these studies highlight both the paucity of comprehensive evidence and the urgent need for culturally adapted interventions tailored to Chinese communities.

Furthermore, existing review studies mainly focus on evidence published in English [13, 14]. The value of Chinese databases has long been overlooked, though recent scholarship has begun to recognise the importance of sources such as the China National Knowledge Infrastructure (CNKI; https://www.cnki.net) [15, 16, 17].

Given the large number of scholars in Chinese communities, a wealth of relevant studies can be anticipated. However, very few reviews have comprehensively incorporated Chinese databases into their analyses. Our mapping review aims to bridge the gap between studies published in Chinese and English by integrating trials involving Chinese participants across these sources, thereby facilitating academic communication in dementia intervention research across diverse cultural contexts. Despite the large number of dementia related RCTs involving Chinese participants, existing reviews have not yet provided a structured overview of the intervention types, care contexts and settings, and outcome domains for existing evidence. This study aimed to systematically map existing RCT evidence on pharmacological and non‐pharmacological interventions for people living with dementia as well as their formal and informal carers within Chinese communities. The key research questions were: (1) What intervention types (pharmacological, TCM, NPI, multicomponent) and combinations have been trialled? (2) In which contexts and settings (geographic location; hospital/community) have trials occurred? (3) Which outcome domains (e.g., cognition, function, QoL, carers) receive priority? By providing a comprehensive mapping of the current evidence landscape, this study offers clinicians and policymakers a clearer foundation for evidence‐informed decision‐making and helps identify priority areas for future research and intervention development tailored to Chinese populations.

## 2 Methods

This systematic review follows the Preferred Reporting Items for Systematic Reviews and Meta‐Analyses (PRISMA) Statement [18]. The study protocol was published elsewhere [19] and registered with the International Prospective Register of Systematic Reviews (PROSPERO, CRD42019134135).

### 2.1 Identification and Selection of Studies

#### 2.1.1 Search Strategy

We used a list of established bilingual searching terms [19] to identify trials of dementia‐related interventions in Chinese communities that were published in either English or Chinese. We first searched for evidence published between January 2008 and December 2018 and then complemented the evidence pool by searching for studies published between January 2019 and June 2020. The starting year of our literature searching aligns with that of a systematic review synthesising dementia evidence in low‐ and middle‐income countries (LMICs), conducted by the *STRiDE* project team (https://stride‐dementia.org/). Given the anticipated volume of publication, we adapted the search strategy [20] from the STRiDE review study for English‐language literature. The Chinese‐language search strategy, due to its specificity and complexity, has been described in detail in a separate publication [19].

#### 2.1.2 Information Sources

For Chinese databases, we used CNKI (https://www.cnki.net) and Wanfang Data (http://www.wanfangdata.com), which are two primary information providers and the largest aggregators of academic digital resources in China. These two platforms provide English interfaces and are accessible globally. We also searched the English bibliographical databases MEDLINE, EMBASE, PsycINFO, CINAHL Plus, Global Health, WHO Global Index Medicus, Virtual Health Library, Cochrane CENTRAL, Social Care Online, BASE, MODEM Toolkit, and Cochrane Database of Systematic Reviews. In addition to database searches, we manually reviewed the reference lists of the identified review studies to supplement the evidence base.

#### 2.1.3 Eligibility Criteria

##### 2.1.3.1 Population

Studies involving individuals aged 18 years or older who were diagnosed with dementia and identified as Chinese by the original study authors were included. We focussed on studies conducted among individuals of Chinese ethnicity or national heritage, regardless of their nationality or location of residence (e.g., in mainland China, Hong Kong, Taiwan or other settings). Studies were excluded if they did not explicitly indicate that the proportion of Chinese subjects exceeded 50%, or if they lacked a specific subgroup analysis focussed on Chinese participants. There were no restrictions on participant characteristics, such as gender, age, or education level, or on the setting, such as their own home, community, clinic, hospital, health centre, or other care settings. Throughout this article, the terms ‘Chinese populations’ and ‘Chinese communities’ are used as pragmatic labels following the terminology in the original trials, and do not imply cultural or sociopolitical homogeneity across regions.

Regarding the types of dementia, we included studies involving individuals diagnosed by a relevant professional with any type and stage of dementia, recognising that a wide range of illnesses and injuries can primarily or secondarily affect the brain and lead to progressive cognitive decline. The types of dementia considered included, but were not limited to, Alzheimer's disease, vascular dementia, frontotemporal dementia, Lewy body dementia, and mixed dementia. We also included studies involving individuals with MCI, mild neurocognitive disorder, or vascular cognitive impairment with no dementia (VCIND), due to their high risk of progressing to dementia [21]. In addition, studies of individuals with co‐occurring conditions alongside dementia or MCI, as well as those with an unspecified dementia or MCI subtype were included as long as a formal diagnosis of dementia or MCI was documented.

The term ‘carers’ refers to individuals involved in provision and management of care, regardless of whether they were paid, resided with the care recipients, or provided direct or indirect care. This includes health and social care professionals, care managers, care workers, administrative staff of care facilities, family carers, other unpaid carers and family members who assist with direct care provision and care decisions.

##### 2.1.3.2 Intervention

From an effectiveness perspective, any type of intervention aimed at improving desired outcomes was eligible, such as pharmacological treatment, non‐pharmacological interventions (NPIs) and multicomponent interventions. For this review, ‘dementia‐related RCTs’ were defined as randomised controlled trials evaluating interventions intended to improve any aspect of health, wellbeing, functioning or quality of life in people living with dementia or mild cognitive impairment, and/or their formal or informal carers. NPIs refers to interventions that do not directly involve medication, including multisensory or occupational therapy, psychotherapy, physical therapy, and cognitive enhancement therapies [22]. Multicomponent interventions are defined as those combining two or more distinct types of interventions (e.g., a pharmacological treatment paired with an NPI.

##### 2.1.3.3 Comparison

Any type of control intervention was eligible, including treatment as usual and alternative treatments.

##### 2.1.3.4 Outcomes

Dementia often triggers complex challenges across a wide range of domains. From the perspective of effectiveness, we accepted any type of outcome that may affect individuals, families, the dementia care workforce, wider society and social or healthcare systems.

##### 2.1.3.5 Study Design and Quality

To identify potential causal relationships, we only included RCTs, thus excluding prospective comparative cohort studies, case‐control studies and cross‐sectional studies. Studies with samples smaller than 50 participants in either the intervention group or comparison group(s) were also excluded to minimise small‐study effects [23, 24].

During full‐text review, studies that failed to provide sufficient information on the randomisation process or used non‐random methods (e.g., alternation, convenience sampling) were excluded. We only included studies that used allocation sequence generation methods assessed as having a low risk of bias according to Version two of the Cochrane risk‐of‐bias (RoB) tool for randomised trials (RoB 2.0) [25]. These methods included computer‐generated random number, random number tables, coin tossing, and similar techniques.

##### 2.1.3.6 Study Selection

**FIGURE 1:**
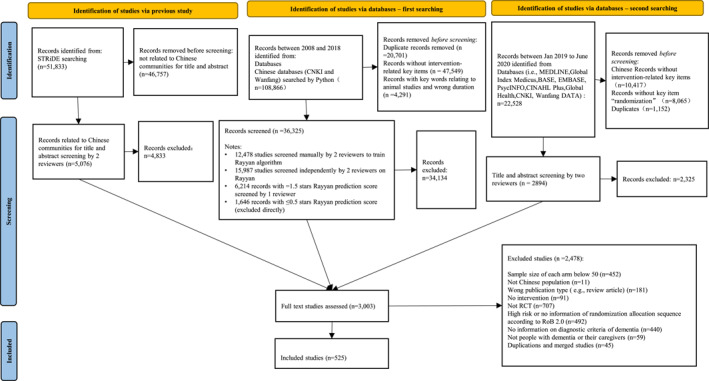
PRISMA flowchart.

Study selection was a two‐step process: (1) title and abstract screening and (2) full‐text review. All reviewers were provided with detailed explanations for inclusion and exclusion criteria in each step. A PRISMA flowchart (Figure 1) shows the process of study selection at each stage.

For studies published between 2008 and 2018 identified from Chinese databases (Figure 1, middle section), due to a large number of records (*n* = 36,325), we employed Rayyan's machine learning‐based classifier to facilitate title and abstract screening. Using a training data set of 12,478 studies manually screened by two reviewers, Rayyan generated a relevance rating for each study, ranging from 0.5 (least relevant) to 5 (most relevant) [26]. We excluded all studies with a score of 0.5. One reviewer screened the studies scoring of 1.5 and two reviewers screened those scoring above 1.5. For studies previously identified by the STRiDE project from English databases (Figure 1, left section) and those published between January 2019 and June 2020 identified from English and Chinese databases (Figure 1, right section), two reviewers independently screened all records.

After the title and abstract screening, the remaining identified studies were uploaded to Covidence for full‐text review by two independent reviewers. Review articles were excluded at this stage, but their reference lists were used to complement the database search results.

Any disagreements at each step were resolved through discussion between the two reviewers. If consensus could not be reached, a third reviewer was consulted to facilitate the final decision.

### 2.2 Data Extraction

We adapted the standardised form on Covidence for data extraction, details of which have been published elsewhere [19]. All data extraction forms were completed by one reviewer and verified by the second reviewer.

### 2.3 Risk of Bias

All 525 included RCTs underwent comprehensive RoB 2.0 [25] assessment by two independent reviewers across five specific domains of bias: (1) bias arising from the randomisation process; (2) bias due to deviations from intended interventions; (3) bias due to missing outcome data; (4) bias in measurement of the outcome; and (5) bias in selection of the reported result. A proposed judgement (‘Low risk', ‘Some concerns', ‘High risk') about the risk of bias is generated by an algorithm, based on answers to the signalling questions for each domain.

### 2.4 Analytic Methods

We used descriptive statistics, crosstabulation and visualisation to map the study trend and focus, and the distribution of RCTs across different geographical regions. Based on the extracted data, data cleaning and descriptive analyses (e.g., frequencies and percentages) were performed using STATA 16.0, by categorising interventions and outcomes according to their nature. The distribution of RCTs was visualised using Microsoft Excel and Flourish (https://flourish.studio), through bar charts, tables, and geographical maps. This analysis was descriptive in nature, focussing on mapping the distribution of RCTs rather than inferring relationships or causality.

## 3 Results

### 3.1 Study Characteristics

The PRISMA flowchart (Figure 1) depicts the systematic process of identifying, screening, and selecting studies for inclusion in this review. Initially, we identified 183,227 studies. After removing duplicates and records without intervention‐related key items, 44,295 studies were screened by title and abstract. Of the remaining 3003 studies subject to full‐text review, 525 unique studies were ultimately included for analysis.

The characteristics of the included studies (*N* = 525) are presented in Table 1. The majority of the trials published in Chinese language (93.1%), while 36 studies (6.9%) were published in English. Dementia types were evenly distributed among Alzheimer's diseases (27.4%), vascular dementia (28.8%), and unspecified dementia (29.1%), with a smaller proportion of participants diagnosed with MCI (14.7%). Detailed characteristics of each included study are presented provided in Table S1.

**TABLE 1:** Characteristics of included RCTs.

	All included studies (*N* = 525)	Chinese‐language publication (*N* = 489)	English‐language publication (*N* = 36)
Setting			
Hospital‐based	91.8%	96.93%	22.22%
Non‐hospital	8.2%	3.07%	77.78%
Funding status			
Funded studies	66.1%	69.1%	25.0%
Studies without available funding information	33.9%	30.9%	75.0%
Dementia type			
Alzheimer's disease	27.4%	28.02%	19.44%
Unspecified dementia	29.1%	28.83%	33.33%
Mild cognitive impairment	14.7%	13.29%	33.33%
Vascular dementia	28.8%	29.86%	13.89%
Overall bias^a^			
Low risk	0.95%	0.00%	13.89%
Some concerns	83.62%	86.09%	50.00%
High risk	15.43%	13.91%	36.11%
Publication language			
Chinese	93.1%	/	/
English	6.9%	/	/

Abbreviation: RCTs = randomised controlled trials.

^a^ Overall bias of included RCTs assessed using the Cochrane risk‐of‐bias tool for randomised trials—version 2 (RoB 2.0).

#### 3.1.1 Trial Contexts and Settings

**FIGURE 2:**
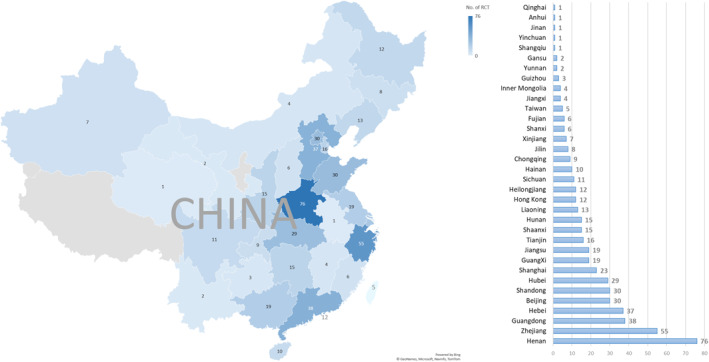
Number of included RCTs by regions. RCTs = randomised controlled trials.

All studies published in Chinese were conducted in mainland China. Half of studies published in English were conducted in Hong Kong (*n* = 12) and Taiwan (*n* = 5), while remaining published in mainland China. No included studies were conducted outside China. Overall, these trials spanned most provincial regions across China (Figure 2). The highest number of RCTs were conducted in Henan (*n* = 76) and Zhejiang (*n* = 55) provinces.

Most trials were hospital‐based (91.8%) and two‐thirds (66.1%) reported a clear funding source. In contrast, among English‐language publications, a much smaller proportion were hospital‐based (22.22%), and only a quarter (25.0%) indicated a funding source.

#### 3.1.2 Type of Interventions

**FIGURE 3:**
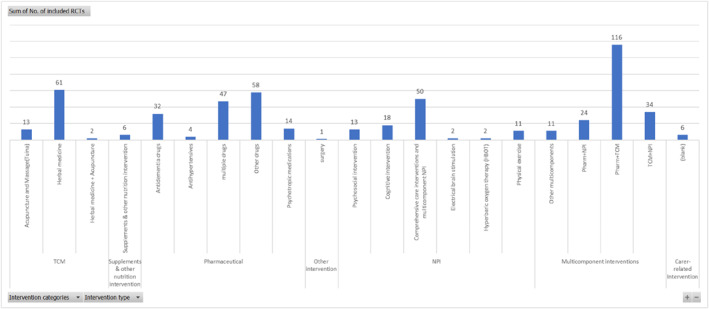
Number of included RCTs by intervention categories (*n* = 525). NPI = non‐pharmacological intervention; RCTs = randomised controlled trials; TCM = traditional Chinese medicine.

We identified seven categories of intervention (Figure 3; Table 2). The largest category was multicomponent interventions (*n* = 185; 35.2%), including 116 studies integrating pharmacological and traditional Chinese medicine (TCM), 34 combining TCM and NPIs, and 24 integrating pharmacological and NPIs. The second‐largest category was pharmacological interventions (*n* = 155; 29.5%), with 58 studies using other drugs, 47 involving drug combinations, 32 using antidementia drugs, 14 involving psychotropics and four using antihypertensives. The category ‘other drugs’ includes drugs not classified as first‐line, officially approved core antidementia agents in mainland China, but it may contain drugs that are used as adjunctive therapy or are researched for their neuroprotective and cognitive‐enhancing properties, particularly in vascular cognitive impairment. Among studies involving NPIs (*n* = 96; 18.3%), 50 examined comprehensive/multicomponent approaches, followed by those focussed on specific modalities like cognitive interventions (18), psychosocial interventions (13), and physical exercise (11), together with electrical stimulation (2) and hyperbaric oxygen (2). TCM interventions (*n* = 76; 14.5%) were also well‐represented, with 61 studies using herbal medicines, 13 focussing on acupuncture or massage, and two combining herbal and acupuncture. Smaller numbers of RCTs were found for supplements and nutrition (6 studies) and other interventions (1 surgery study). Only six studies involved carer‐related interventions.

**TABLE 2:** Number of included RCTs by target outcomes.

Intervention category	Total included RCTs		Included RCTs by target outcome						
			Cognition	Functioning	Behaviour	Memory	Quality of life	Depression	Carer‐related outcomes
	*N*	%	%	%	%	%	%	%	%
Multicomponent interventions	185	35.2%	87.6%	58.4%	3.8%	4.9%	1.6%	0.5%	0.0%
Pharmacological	155	29.5%	89.0%	64.5%	8.4%	2.6%		0.6%	0.1%
NPI	96	18.3%	81.3%	57.3%	3.1%	5.2%	12.5%	7.3%	0.1%
TCM	76	14.5%	93.4%	63.2%	7.9%	2.6%	0.0%	1.3%	0.0%
Carer intervention	6	1.1%	16.7%	16.7%	33.3%	0.0%	0.0%	50.0%	16.7%
Supplements and other nutrition intervention	6	1.1%	83.3%	50.0%	0.00%	0.0%	0.0%	0.0%	0.0%
Surgery	1	0.2%	100%	100%	0.00%	0.0%	0.0%	0.0%	0.0%
Total	525	100%	86.9%	60.2%	5.9%	3.8%	2.9%	2.5%	0.7%

*Note:* Each intervention may target multiple outcomes. All target outcome.

Abbreviations: NPI = non‐pharmacological intervention; RCTs = randomised controlled trials; TCM = traditional Chinese medicine.

#### 3.1.3 Combination in Multicomponent Intervention

**FIGURE 4:**
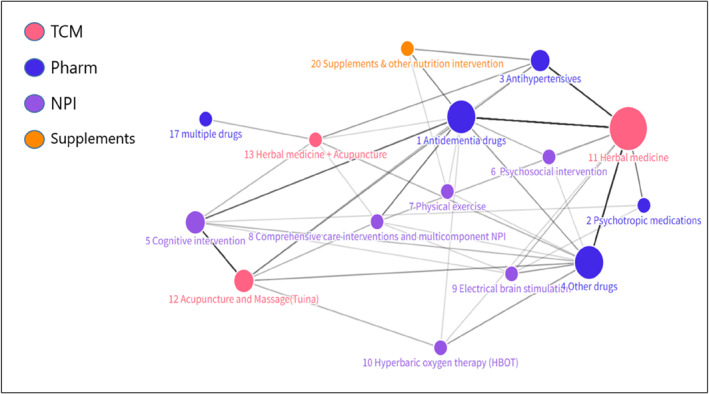
Combination of multicomponent interventions (*n* = 185). Each intervention category is assigned a different colour in the figure, with the size of the colour indicating the number of RCTs using that intervention. The lines connecting interventions show the combinations observed in the RCTs, with the darkness representing the frequency of those combinations. This figure is generated by Flourish (https://flourish.studio). NPI = non‐pharmacological intervention; RCTs = randomised controlled trials; TCM = traditional Chinese medicine.

Figure 4 provides a visualisation of the intervention combinations among the 185 RCTs involving multicomponent interventions. All possible combinations across intervention categories (i.e., TCM, Pharm, NPI, and Supplements) were observed. TCM and pharmacological interventions were the most frequently represented, indicating their common use in conjunction with other interventions. Among specific types of interventions, the most common combination was antidementia drugs and herbal medicine (i.e., combination of type 1 and 11 in Figure 4).

#### 3.1.4 Target Outcomes

Overall, the included RCTs mainly focussed on improving cognitive and functional outcomes of individuals living with dementia, with less attention given to behaviour (5.9%), memory (3.8%), QoL (2.9%), depression (2.5%) or carer‐related outcomes (0.7%; Table 2). The included RCTs focussing on different intervention categories also showed similar pattern of target outcomes, that is, primarily focussing on improving cognition and enhancing functioning.

#### 3.1.5 Study Quality

**FIGURE 5:**
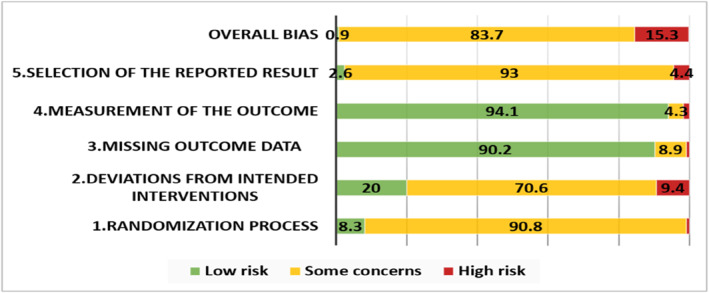
Risk of bias of included RCTs assessed using the Cochrane risk‐of‐bias tool for randomised trials—version 2 (RoB 2.0).

The majority of included studies were assessed as having ‘some concerns’ in the domains of the randomisation process, deviations from intended interventions, and selection of reported results, while showing ‘low risk’ in missing outcome data, and measurement of outcome (Figure 5 & Table 1). Overall, over 80% of studies were rated as having ‘some concerns’, indicating common risks of bias related to inadequate concealment of the allocation sequence, uncertainties in the blinding of participants, interventionist, and/or research personnel, and potential selective reporting of analyses and outcomes. Moreover, 15% of studies were assessed as having ‘high risk’ whereas fewer than 1% were rated as ‘low risk’. None of the studies published in Chinese were low risk, with most (86.09%) raising some concerns, and 13.91% being high risk. These studies published English demonstrated a higher proportion of low‐risk assessments (13.89%) and a lower rate of some concerns (50.00%), though more than one‐third (36.11%) were still considered high risk.

## 4 Discussion

This study represents the first bilingual systematic review to comprehensively map dementia interventions in Chinese and English literature involving participants identified as Chinese, addressing a critical gap in synthesising global evidence. By employing dual‐language screening teams and culture‐sensitive searching terms, we maximised selection comprehension while capturing region‐specific interventions such as acupuncture and herbal formulations. Following PRISMA guidelines, we synthesised 525 RCTs from an initial pool of 183,227 records, revealing critical insights into the evolving dementia intervention landscape in Chinese communities. Evidence on the effectiveness of interventions for individuals living with dementia in Chinese communities is growing rapidly, particularly within Chinese‐language publications. All included studies were conducted in mainland China, Hong Kong and Taiwan, and none were carried out in ethnic Chinese communities outside of these regions, indicating that the mapped evidence primarily reflects intervention research within Chinese health and social care systems rather than the broader global Chinese diaspora.

First, we found that RCTs have been conducted among Chinese population for a wide range of intervention categories, including multicomponent interventions (*n* = 185; 35.2%), pharmacological interventions (*n* = 155; 29.5%), NPIs (*n* = 96; 18.3%) and TCM (*n* = 76; 14.5%). Among multicomponent trials, the combination of TCM and pharmacological intervention was the most prevalent (62.7%). Second, most RCTs focussed on improving cognitive and functional outcomes, with limited attention to QoL, behaviour, and carer‐related outcomes. Also, no studies reported economic outcomes. Third, in terms of geographical distribution and trial settings, most trials were concentrated in urban areas and hospital settings in mainland China, with limited representation from rural regions, non‐hospital settings and oversea Chinese communities. Last, most trials were assessed as having ‘some concerns’ in their blinding and randomisation processes, which may potentially inflate their efficacy estimates.

### 4.1 Comparison With Global Evidence

Previous large‐scale mapping reviews have laid important groundwork for understanding dementia interventions globally. The *MODEM* project [27] systematically mapped English‐language evidence from five databases (MEDLINE, PsychINFO, CINAHL, Social Care Online, and IBSS) covering publications from 2009 to 2015, and made these evidence accessible through a searchable bibliographic database (https://toolkit.modem‐dementia.org.uk). Since the interventions identified were primarily conducted in high‐income countries, building on this effort, the *STRiDE* project (https://stride‐dementia.org/) expanded the scope by reviewing 12 databases (MEDLINE, EMBASE, PsycINFO, CINAHL Plus, Global Health, WHO Global Index Medicus, Virtual Health Library, Cochrane CENTRAL, Social Care Online, BASE, MODEM Toolkit, Cochrane Database of Systematic Reviews) to capture evidence from low‐ and middle‐income countries (LMICs) between 2008 and 2018. However, that review still included mostly English language studies and it did not include targeted searches in Chinese databases [5]. While these reviews identified China as the source of 69.7% of LMIC dementia trials [5], their reliance on Anglo‐centric databases likely underrepresented culturally specific intervention, such as TCM, that are commonly documented in Chinese journals. Our study advances this field by incorporating *CNKI* and *WanFang DATA*—two comprehensive Chinese databases—alongside the 12 databases used in STRiDE. This enabled the first truly bilingual synthesis of dementia interventions across Chinese communities. Our approach addressed critical gaps in prior reviews by capturing regionally prevalent but linguistically siloed evidence, particularly for culturally embedded practices such as TCM, NPI, integrative multicomponent interventions and carer‐focused interventions [19].

While a growing body of systematic reviews has evaluated dementia interventions, most synthesise evidence disproportionately represented Western and high‐income country perspectives. The linguistic bias obscures culturally specific interventions prevalent in LMICs. The STRiDE review study found that the largest category of dementia‐related interventions studied in LMICs was TCM (43.8%), followed by Western pharmacological (32.1%) and supplements (12.6%) [5], and the majority of RCTs were conducted in China (69.7%). Our bilingual review extends these findings by revealing that multicomponent interventions dominate the Chinese evidence base, with approximately two‐thirds combining TCM (e.g., herbal formulations, acupuncture) with Western drugs. This integrative approach reflects a unique policy‐driven healthcare landscape in mainland China. The Chinese government's longstanding promotion of *integrated Traditional Chinese and Western medicine* has incentivised hybrid intervention designs. Our analysis identified a broader spectrum of RCT‐verified TCM interventions that were evident from prior English‐language reviews, including herb‐drug combinations and non‐pharmacological therapies such as acupuncture. Clinically, this preference may stem from cultural beliefs that TCM addresses holistic health (e.g., *qi* balance) while Western drugs target specific symptoms—a dual‐pathway model aligning with recipients' expectations in Chinese communities.

Our review identified a more limited repertoire of NPIs in Chinese communities compared to Western literature. While comprehensive multidomain interventions predominate in China, particularly mainland China, several evidence‐based NPIs that are commonly implemented in Western settings [28]—particularly empowerment‐based approaches—remain underrepresented in Chinese trials. This disparity appears driven by three key factors: (1) significant funding disparities favouring pharmacological research; (2) a hospital‐centric research infrastructure ill‐suited for many NPIs, and (3) the nascent state of China's long‐term care system. Notably, 97% of included Chinese‐language studies were conducted in hospital settings, while community‐based interventions, which dominate Western NPI research, accounted for only 12% of Chinese trials. This institutional misalignment is further compounded by the medical insurance policies in mainland China which currently reimburse hospital‐based care but provide less cover for community or home‐based interventions [29]. These systemic constraints have likely delayed the development and evaluation of NPIs that are best delivered in community settings, creating a distinctive intervention landscape that prioritises medically supervised, structured programmes over the person‐centred approaches more prevalent in Western dementia care.

Furthermore, our review also reveals a striking neglect of carer‐focused interventions, despite the projected increase in the nation's informal dementia carers to 49 million by 2060 in mainland China [30]. Remarkably, we found that only 1% of included RCTs targeted carer outcomes, a pattern consistent with broader LMIC trends where fewer than 5% of dementia trials address carer well‐being [5]. This oversight persists despite strong global evidence supporting the efficacy of carer interventions [31], including: (1) psychoeducational programmes that reduce the caregiving burden through skill‐building, and (2) psychotherapeutic approaches like Cognitive Behavioural Therapy (CBT) and Acceptance and Commitment Therapy (ACT) effectively mitigate carer anxiety and depression. While small‐scale trials of such interventions may exist in China, they remain largely unpublished or methodologically limited. Even when interventions involve carers, outcome measures typically focus on the benefits to the individual living with dementia rather than their carer. This systemic inattention to carer needs reflects both research priorities and structural barriers in China's care system, where carer support lacks institutional recognition and funding mechanisms [14, 32].

Despite its fundamental importance in person‐centred dementia care, QoL remains strikingly understudied as a primary outcome in Chinese intervention research. While QoL encompasses multidimensional well‐being across physical, psychological, social, and environmental domains [33], very few included trials specifically targeted QoL outcomes. This oversight persists despite the availability of culturally adapted assessment tools such as the EQ‐5D [34] and QoL‐AD Chinese versions. Notably, existing interventions predominantly prioritise clinical biomarkers and cognitive metrics over subjective well‐being measures, reflecting a persistent biomedical orientation in Chinese dementia research. The paucity of QoL‐focused studies represents a critical gap, as it limits understanding of how interventions affect the lived experiences of individuals living with dementia.

### 4.2 Study Quality

The methodological rigour of the included trials warrants cautious interpretation of efficacy claims. The RoB 2 assessment resulted in a substantial proportion of studies (over 80%) being rated as subject to ‘some concerns’, primarily due to inadequate blinding procedures and poorly documented randomisation methods (over 90%; e.g., failure to specify allocation concealment). It implies that many studies, particularly those published in Chinese journals, may not have fully adhered to international reporting standards (e.g., CONSORT guidelines), omitting critical methodological details. Also, certain interventions (e.g., acupuncture) face practical difficulties in standardisation and blinding, which may also contribute to methodological constraints. English‐language publications demonstrated a somewhat more robust methodological profile, with a notable proportion (13.89%) achieving ‘Low risk’. However, it is important to note that over one‐third (36.11%) were still deemed ‘High risk’, underscoring that bias remains a significant issue even in internationally accessible literature.

### 4.3 Geographical Distribution and Trial Settings

Our review identified a complete absence of RCTs conducted among ethnic Chinese diaspora communities outside of China, limiting the generalizability of findings to these culturally similar but contextually distinct populations. Among English‐language publications (7%), half of studies were conducted in Hong Kong and Taiwan, while remaining half were carried out in mainland China.

Included RCTs were predominantly concentrated in urban areas and hospital settings in mainland China, with limited representation from rural regions and community settings. This urban skew neglects rural communities, where fragmented healthcare infrastructure and lower health literacy may significantly impact the feasibility of interventions and outcomes [35, 36]. The paucity of non‐hospital‐based trials among Chinese‐language publications (only 3%) also obscures real‐world effectiveness, as hospital settings often fail to replicate the physical environments and social contexts shaping dementia care in daily life. Collectively, these limitations underscore an urgent need for rigorously designed, pragmatically oriented RCTs that prioritise blinding protocols, transparent randomisation, and equitable geographical and socioeconomic representation to strengthen evidence‐based practice.

### 4.4 Implications for Practice and Research

The findings from this systematic review highlight several critical directions for future dementia research. First, there is an urgent need to rebalance research priorities by increasing funding and methodological support for studies on NPIs, carer support, and QoL outcomes among Chinese population. Given the demonstrated efficacy of these approaches in global contexts, researchers should prioritise pragmatic trials that evaluate interventions in real‐world community settings rather than relying on hospital‐based studies. Second, a significant evidence gap exists from well‐established oversea Chinese communities, such as those in Singapore and the United State, and the other regions with large Chinese population. It is possible that the varying health system structures and evolving social support networks in these contexts may modify the effectiveness of interventions developed within mainland China. Addressing this gap is essential for building a truly generalisable evidence base that reflects the diversity of Chinese communities worldwide. Third, recognising the family as a unit of care moves beyond a patient‐centric or dyadic approach acknowledge that dementia care unfolds within an interdependent system [37]. In Chinese communities, family caregiving is deeply embedded. Future studies should prioritise the interventions for improving dementia caregivers' outcomes and interrelationship between the outcomes of persons with dementia and their caregivers.

Based on the substantial body of evidence from mainland China, we also propose the following policy implications situated within this context. Specifically, healthcare policymakers can expand medical insurance coverage to include community‐based and home‐delivered interventions [38], thereby incentivising the development of NPIs outside hospital settings. Training programmes for healthcare professionals should integrate carer support strategies, including psychoeducation and cognitive‐behavioural techniques, to address the high levels of psychological distress among carers of people living with dementia. Additionally, China's long‐term care infrastructure must be strengthened to support intervention delivery, particularly in rural and underserved regions. Given the government's emphasis on integrated care models, dementia interventions should be incorporated into primary healthcare systems with a focus on holistic, person‐centred outcomes. By addressing these research and systemic gaps, the Chinese government can develop a more comprehensive, evidence‐based dementia care framework that aligns with global standards while meeting local needs.

### 4.5 Limitations of the Present Study

Several limitations of this study should be acknowledged. First, while we identified key interventions for the target population, we did not systematically synthesise and compare their effectiveness, which limits our ability to draw definitive conclusions about optimal approaches. To better understand the effectiveness of these interventions, studies using pairwise and network meta‐analyses are needed. These more advanced analytical techniques can provide more nuanced insights into the comparative effectiveness of different interventions, guiding future research and policy decisions. Second, our focus on RCTs may have excluded other intervention studies conducted in Chinese communities, such as quasi‐experimental or qualitative studies. However, we prioritised RCTs as they provide the most robust level of evidence. Third, to ensure methodological rigour, we imposed a minimum sample size requirement (≥ 50 participants per study arm), which may have excluded smaller‐scale interventions with potential relevance. Given the variability in study quality, future reviews could apply additional filters, such as restricting analyses to peer‐reviewed journals with higher impact factors, to further strengthen the validity of findings. While shared cultural values, beliefs, and genetic characteristics across Chinese populations may influence dementia care and intervention outcomes, it is also critical to acknowledge the heterogeneity within the Chinese communities. However, given that the overwhelming majority of exiting evidence were generated from mainland China, with few evidence form oversea Chinese communities, this heterogeneity remains underexplored.

## 5 Conclusions

This mapping review provides a comprehensive understanding of current research on dementia interventions involving Chinese participants, where the prevalence of dementia is notably high. It reveals a diversity of interventions trialled, particularly those documented in Chinese‐language publications, and identifies key research gaps in terms of intervention type and target outcomes. With a rapidly expanding evidence base, our study also serves as a valuable example of managing large‐scale systematic reviews. The methodological rigour employed in our study, including dual‐language screening teams and culture‐sensitive inclusion criteria, ensures that the findings are robust and reliable. By promoting international knowledge exchange, supporting policy implementation, and highlighting gaps in carer interventions, our study contributes to the ongoing efforts to improve dementia care worldwide.

## Funding

This work was conducted as part of the ‘Tools to Inform Policy: Chinese Communities Actions in Response to Dementia’ (TIP‐CARD) project, supported by the Hong Kong Research Impact Fund of the Research Grants Council (Project Reference No.: R7017‐18). MSK, DMD, MK and ACH's contributions were supported by the UK Research and Innovation's Global Challenges Research Fund (ES/P010938/1) as part of the ‘Strengthening Responses to Dementia in Developing Countries’ (STRiDE) project.

## Conflicts of Interest

The authors declare no conflicts of interest.

## Supporting information


**Table S1:** Characteristics of each included study.

## Data Availability

This systematic review was conducted using data derived exclusively from previously published studies. No primary data collection was performed. The search strategy, inclusion criteria, and list of included studies are provided in the manuscript and supplementary materials. Given the nature of the review, no additional datasets were generated or analysed beyond what is explicitly reported here.
